# Oxidative stress in genetically triggered thoracic aortic aneurysm: role in pathogenesis and therapeutic opportunities

**DOI:** 10.1080/13510002.2021.1899473

**Published:** 2021-03-13

**Authors:** Stefanie S. Portelli, Brett D. Hambly, Richmond W. Jeremy, Elizabeth N. Robertson

**Affiliations:** a Discipline of Pathology and Charles Perkins Centre, The University of Sydney, Sydney, Australia; b Cardiology Department, Royal Prince Alfred Hospital, Sydney, Australia

**Keywords:** Thoracic aortic aneurysm, aortic dilatation, aortic dissection, Marfan syndrome, Bicuspid Aortic Valve, ROS, oxidative stress, redox

## Abstract

**Background:** The primary objective of this review was to explore the contribution of oxidative stress to the pathogenesis of genetically-triggered thoracic aortic aneurysm (TAA). Genetically-triggered TAAs manifest substantial variability in onset, progression, and risk of aortic dissection, posing a significant clinical management challenge. There is a need for non-invasive biomarkers that predict the natural course of TAA and therapeutics that prevent aneurysm progression.

**Methods:** An online systematic search was conducted within PubMed, MEDLINE, Scopus and ScienceDirect databases using keywords including: oxidative stress, ROS, nitrosative stress, genetically triggered thoracic aortic aneurysm, aortic dilatation, aortic dissection, Marfan syndrome, Bicuspid Aortic Valve, familial TAAD, Loeys Dietz syndrome, and Ehlers Danlos syndrome.

**Results:** There is extensive evidence of oxidative stress and ROS imbalance in genetically triggered TAA. Sources of ROS imbalance are variable but include dysregulation of redox mediators leading to either insufficient ROS removal or increased ROS production. Therapeutic exploitation of redox mediators is being explored in other cardiovascular conditions, with potential application to TAA warranting further investigation.

**Conclusion:** Oxidative stress occurs in genetically triggered TAA, but the precise contribution of ROS to pathogenesis remains incompletely understood. Further research is required to define causative pathological relationships in order to develop therapeutic options.

## Introduction

Thoracic aortic aneurysm (TAA) is a clinically silent phenomenon, with subsequent aortic dissection the leading cause of morbidity and early mortality. Genetically triggered TAA accounts for 30% of all TAA pathology, which presents either in the context of a clinically recognisable syndrome: Marfan (MFS), Loeys Dietz (LDS), vascular Ehlers-Danlos (vEDS), or in individuals with no syndromic features: Bicuspid Aortic Valve (BAV), and familial Thoracic Aortic Aneurysm and Dissection (fTAAD). Further details can be found in The National Registry of Genetically Triggered Thoracic Aortic Aneurysms and Cardiovascular Conditions (GenTAC) [1].

All genetically triggered TAAs demonstrate heterogeneity in clinical severity, indicating a complex multifactorial pattern of disease that remains unclear. Aneurysm size is a poor indicator of dissection risk, with dissections occurring at normal aortic diameters in some patients and TAAs stabilising in others [2, 3]. However, given aortic dissection carries a mortality rate of up to 50% [4], prophylactic surgery is usually performed if the aorta progressively dilates, as no current medical treatments stop or reverse the dilatation [5]. Surgery still carries a mortality risk of 1-5% for elective repair [6] and up to 12% for emergency intervention [7]. Therefore, there is a need for improved understanding of the pathomechanics of TAA formation to improve detection and management.

Aortic dilatation, aneurysm and dissection are, at their core, a varying continuum of manifestations of a central biomechanical failure. Dysfunctional protein interactions and signalling within the aortic wall lead to failed mechanotransduction, namely the ability of intramural cells to sense their mechanical environment and produce appropriate biochemical responses [8]. Thus, repair and restorative mechanisms that continually maintain wall homeostasis become dysfunctional, causing medial degeneration, characterised by extracellular matrix (ECM) accumulation and proteolytic degeneration, vascular smooth muscle cell (VSMC) phenotype switching and apoptosis [9]. Compounded by the large cyclical haemodynamic pressures in the aortic wall, particularly greatest in the aortic root and ascending aorta, localised weakness results in dilatation and aneurysm formation [10]. This is evident in the pathogenic gene variants that give rise to TAA, which all have structural and/or functional roles in aortic wall homeostasis. These include genes that encode proteins responsible for (i) ECM regulation (*FBN1*, *COL3A1, LOX, MFAP5, BGN*), (ii) the VSMC contractile apparatus (*MYH11*, *ACTA2, MYLK, FLNA, PRKG1*), or (iii) transforming growth factor-beta (TGF-β) signalling (*TGFB2, TGFB3, TGFBR1, TGFBR2*, *SMAD3*) [11].

## A pro-oxidant environment

**Oxidative stress** and nitrosative stress occur from imbalances in the production and clearance of reactive oxygen species (ROS) and reactive nitrogen species (RNS), which are highly reactive free radicals (a molecule with one or more unpaired electrons in its outer shell) that are capable of damaging all cellular constituents including DNA, proteins, and lipids, resulting in loss of function and tissue injury [12]. ROS and RNS are natural by-products of aerobic metabolism and oxidative enzymes, and under normal conditions are continually eliminated or neutralised by antioxidant defences. Low levels of ROS/RNS are essential for physiological homeostasis, while excess levels – either through increased generation or insufficient removal – are widely implicated in disease [13].

**Biomarkers** that are specific for oxidative and nitrosative stress are useful for characterising redox pathways in disease, including the oxidant source, specific effects on tissue, and assist in developing candidate targets for therapeutics. For example, isoprostane, malondialdehyde and oxidised low-density lipoprotein (ox-LDL) levels are markers of lipid peroxidation, while nitrotyrosine, chlorotyrosine, carbonylation and S-glutathionylation are products of oxidative protein modifications. Alternatively, evaluation of redox status may be determined by gene and protein levels of redox enzymes [14, 15].

In genetically triggered TAA, oxidative stress has been widely demonstrated in aortic tissue from animal models [16] and patients with MFS [17–20], BAV [20–25], LDS [26] and in multi-phenotype TAA cohorts [27]. In the aortic wall, oxidative stress disrupts mechanosignalling [28], promotes expression of ECM-degrading matrix metalloproteinases (MMP), and induces pathological VSMC phenotypic switching and apoptosis, which all precipitate aortic wall degeneration and TAA formation [29]. Human TAA subjects have higher peak wall stress, which correlates with histological observations of ROS accumulation and pathological synthetic VSMC populations [30].

In MFS, which is defined by pathogenic variants in *FBN1,* the relationship between oxidative stress and TAA is likely due to altered biomechanics resulting from abnormal fibrillin-1 protein product [31]. Fibrillin-1 contributes to elastic fibre formation throughout the body, and importantly, the aortic wall. The normally elastin-rich aortic wall is therefore intrinsically weakened from birth leading to TAA and/or dissection as early as childhood [32]. While the pathophysiology of TAA in MFS is still unclear, aberrant TGF-β signalling is known to occur and is purported to cause disrupted ECM homeostasis precipitating aortopathy [33].

Recent data have shown a role of oxidative stress in the pathogenesis of MFS, although the source and contribution of ROS are unknown. Plasma from MFS patients is deficient in total antioxidant capacity (TAC), which correlates with clinical severity, consistent with a more extensive contribution of oxidative stress in the multisystem manifestations of MFS [34]. Studies using MFS mice have demonstrated impaired aortic contraction and relaxation of the aorta *in vivo,* with increased lipid peroxidation, increased pro-oxidant enzyme expression and decreased expression of the major ROS-clearing antioxidant, superoxide dismutase (SOD), within the aortic wall [16]. Furthermore, increased ROS production has shown to be limited only to the aneurysmal site and not extending to normal aortic tissue distally [35]. Importantly, restoration of normal redox homeostasis with respective treatments (ROS inhibition or antioxidant supplementation) improved vasomotor function and attenuated aneurysmal dilatation, providing scope for ROS as a future therapeutic target.

BAV is a complex cardiovascular condition characterised by abnormal fusion of aortic valve cusps resulting in a two-cusped valve of variable morphology and function [36]. The aetiology is unknown but at least partly gene-mediated given its strong inheritance patterns. TAA occurs in up to 45% of patients with BAV and the pathomechanics are similarly unexplained but are thought to associate with altered haemodynamics arising from the abnormal valve, leading to asymmetrically increased wall stress [37, 38]. Advances in imaging over the last decade have demonstrated the altered flow dynamics in BAV-TAA, with further research indicating an association with oxidative stress [37, 39, 40]. Paradoxically, levels of the superoxide anion (O_2-_) are significantly greater in aortic non-aneurysmal vs aneurysmal human BAV specimens, suggesting a more prominent role for ROS in early aortopathy and that the characteristic VSMC loss may be a direct consequence of ROS-mediated cell damage [21].

An increase in connective tissue growth factor (CTGF) expression in TAA has been associated with excess ROS. CTGF, a member of the TGF-β family, is increased in both human and mouse TAA tissue [30], with a positive correlation between TAA diameter and CTGF mRNA and protein expression, in addition to osteopontin, a marker of synthetic VSMC phenotype [41], supporting earlier studies which showed increased collagen and CTGF expression in dissected TAA aortic specimens [42]. Furthermore, cultured aortic VSMCs treated with hydrogen peroxide (H_2_O_2_) had ROS-induced VSMC phenotype switching from contractile to synthetic, mediated through CTGF [30], supporting its role in ECM synthesis and VSMC proliferation via ROS-mediated oxidative damage.

## Sources of ROS imbalance

### Insufficient ROS removal

The human host is equipped with a three-tier system of antioxidant defences for efficient ROS/RNS removal [43]. Tier 1 are the small molecule antioxidants which directly scavenge ROS/RNS to prevent the initiation of oxidative stress: the glutathione (GSH) system, metallothionein (MT), uric acid, and vitamins C and E. Tier 2 are the antioxidant enzymes which detoxify RNS/RNS into less reactive species: superoxide dismutases (SOD), catalase, glutathione peroxidases (GPx) and peroxiredoxins (PRX). Tier 3 are enzymes involved in damage control and repair.

Impaired functioning of the **GSH** system occurs in both MFS and LDS, with low GSH observed in TAA tissue in concert with decreased total antioxidant capacity, increased lipid peroxidation and increased carbonylation [26, 44]. The GSH system, involving glutathione peroxidase (GPx), glutathione S-transferases (GST), and glutathione reductase (GR), is the most important and abundant small molecular weight antioxidant in cells with myriad protective functions in addition to neutralising oxidative stress [45, 46]. The ratio of glutathione in its reduced (GSH) to oxidised (GSSG) state is a recognised indicator of local redox status, with a low ratio indicating oxidative stress [47]. Loss of GSH homeostasis as a key primary defence against ROS/RNS results in endothelial and smooth muscle cell dysfunction, and is implicated in many chronic degenerative diseases across multiple body systems [48]. The mechanisms of its dysregulation are an area of active research, with hope for utilisation of GSH as a biomarker or pharmacological target [49].

A reduction in **MT** gene and protein expression has been observed in BAV-TAA tissue and isolated VSMC cultures in concert with increased aortic MMP-9 expression [24]. Altered MT expression is also described in multiple pathologies including cardiovascular disease, diabetes, obesity, renal and liver toxicity, and carcinogenesis [50]. MTs are metal-binding antioxidant proteins with diverse biological functions including heavy metal detoxification, MMP regulation and scavenging of free radicals [51], thus low levels may contribute to MMP-mediated ECM degradation. While inducers of MTs are established and include ROS among other stimuli [51], suppressors of MT expression are less well known [52] but at least include gene silencing by DNA methylation [53]; alternatively, reduced levels might reflect an impaired capacity of damaged cells to induce MT expression [24].

Reduced levels of **SOD** and **catalase** have been observed in aortic media homogenates from dissected TAAs in concert with increased lipid peroxidation [54]. Similar results have been observed in murine MFS-TAA studies [16]. Meanwhile, regional variability in the expression of SOD isoforms was shown in BAV-TAA tissue, which corresponded to aortic diameter and aortic wall segment [22]. The regional differences in BAV-TAA cohorts demonstrate particularly well the notion of oxidative stress as an intermediary between haemodynamic stress and aortic dilatation. Indeed, certain subgroups of BAV-TAA with comorbid aortic stenosis have eccentric flow jets that regionally correspond with wall shear stress in the ascending aorta [2]. Importantly, overexpression of both SOD and catalase have been shown to be cardioprotective [43]. Given a wide range of cytokines and growth factors are capable of altering SOD expression [55], further studies that elucidate the upstream source of dysregulation will be useful.

While fewer studies have specifically examined catalase in genetically triggered TAAs, a putative role is suggested from research in abdominal aortic aneurysms (AAA). In murine AAA models, studies have shown a loss of catalase in aortic tissue sections, while catalase overexpression prevented early pathological wall remodelling [56] and inhibited AAA formation [57].

### Excess ROS generation

Virtually all cells within the vessel wall are capable of ROS/RNS generation [13]. These include the enzymic sources: NADPH oxidase (NOX), xanthine oxidase (XO), myeloperoxidase (MPO), lipoxygenase (LOX), cyclooxygenase (COX), uncoupled endothelial nitric oxide synthase (eNOS), other amine oxidases, and non-enzymic sources including electron leakage from the mitochondrial electron transport chain. ROS are also produced from the endoplasmic reticulum (ER) during ER stress, as the ER is highly sensitive to the local redox status and changes to both extracellular and intracellular homeostasis [58].

Evidence of **mitochondrial dysfunction** leading to reduced mitochondrial respiration and ROS is speculated to have a role in genetically triggered TAA pathogenesis, with existing associations established in AAA, cardiovascular disease and normal vascular ageing [59], but so far limited research in TAA cohorts. A *fibulin-4^R/R^* murine model of genetically triggered TAA demonstrated altered mitochondrial protein composition and decreased oxygen consumption in concert with increased ROS, with genomic studies showing a dysregulation of metabolic pathways [60]. Fibulin-4 is a structural glycoprotein found in the aortic media, necessary for integrity and elasticity of the aortic wall [61]. The mitochondrial electron transport chain constitutively secretes ROS as a by-product of normal aerobic respiration which is cleared under redox homeostasis [13]. However, excess ROS in the local environment can cause oxidative damage to mitochondrial DNA, leading to further ROS production and potentially a vicious cycle of ROS-induced ROS damage [59].

Increased **NOX4** expression and tyrosine nitration were observed in the aortic wall and cultured VSMCs of MFS-TAA patients, which corresponded with increased H_2_O_2_ production and oxidative damage to multiple cytoskeletal and contractile proteins [17]. The involvement of NOX4 in TAA was also examined in a murine NOX4-deficient MFS mouse model which showed less aortic root dilatation and elastic fibre degradation at nine months [17]. NOX is the major ROS producer in the vasculature and increased levels are established in a range of pathological conditions including hypertension, diabetes and hypercholesterolaemia [13]. NOX inhibitors are under intensive investigation for therapeutics [62–64], especially given their sole function is ROS production unlike other sources which produce ROS as by-products or only under stress [64].

**Xanthine oxidase** (XO) was shown in murine MFS-TAA models to be a major contributor of ROS production leading to impaired aortic contraction and relaxation, as reversal of these effects were demonstrated by selective XO inhibition [16]. XO is localised to endothelial cells, and catalyses oxidation of hypoxanthine and xanthine, producing superoxide and H_2_O_2_ as by-products [65]. Increased XO activity and oxidative stress are also demonstrated in coronary artery disease [65] and ruptured cerebral aneurysm [66]. Notably, oscillatory shear stress is an inducer of XO [67], as well as ECM degrading enzymes that trigger aortic wall remodelling [68], therefore, it is biomechanically possible that deranged haemodynamics may precipitate ROS-induced TAA development.

**SmgGDS** (Small GTP-Binding Protein GDP Dissociation Stimulator) is also implicated in TAA pathogenesis due to decreased expression in human TAA tissue [69]. Among other roles, SmgGDS maintains VSMCs in the contractile phenotype necessary for normal aortic function [69, 70]. In a SmgGDS-deficient TAA mouse model there was more severe aortic dilatation and more extensive elastin fragmentation, higher levels of ROS, MMPs and inflammatory cell migration, with dilatation reversed by delivery of a *SmgGDS* gene construct. Cultured human aortic VSMCs deficient in SmgGDS also showed decreased expression of multiple contractile genes, further supporting a role for SmgGDS in maintenance of aortic function [70, 71].

Elevated levels of the pro-inflammatory enzyme **myeloperoxidase** (MPO) are widely implicated in the pathogenesis of AAA [72–74], intracranial aneurysms [75–77], in other cardiovascular diseases [78], and in inflammation across multiple body systems [79]. MPO, a haem peroxidase, is produced predominantly by neutrophils [80], and its expression in vascular inflammation is localised to the endothelium and sub-endothelial space [78]. MPO catalyses a unique conversion of H_2_O_2_ to the highly reactive hypochlorous acid (HOCl). High levels of HOCl cause vascular damage through multiple pathways including lipid and protein oxidation [81], reduced eNOS stability and NO production, causing impaired vasorelaxation [82, 83], and increased MMP activation causing ECM degradation [78], together contributing to aneurysm formation.

Key markers of MPO-mediated oxidative damage via excessive HOCl activity are the generation of 3-nitrotyrosine and 3-chlorotyrosine protein modifications [81]. Activation of the extracellular signal-regulated kinase (ERK) 1/2 has been correlated with both 3-nitrotyrosine and 3-chlorotyrosine modifications, and promotes human aortic VSMC migration [84, 85], consistent with the pathological synthetic phenotype associated with TAA pathogenesis [86].

Increased MPO has been observed in aortic wall specimens from a mixed cohort of TAA patients [72]. Additionally, a MFS mouse model showed increased MPO expression in the aneurysmal aorta, along with increased MMP-2 and -9 expression, increased ECM fragmentation and apoptosis, increased 3-nitrotyrosine levels and increased ROS staining compared to wildtype [87]. Conversely, MPO-deficient MFS mice had no markers of ROS damage or MMP overexpression, preserved aortic architecture and smaller aneurysmal diameter. In a small study of human BAV-TAA, increased plasma MPO correlated with increasing valve and endothelial dysfunction, but did not correlate with TAA severity, however, this may be due to lack of statistical power [88].

### Endothelial dysfunction, Nitric Oxide & NOS enzymes

Endothelial dysfunction is a major contributor to aneurysm formation [89] and is observed in MFS-TAA [90, 91], AAA [92] and cardiovascular disease more broadly [93]. Endothelial cells modulate vascular homeostasis through multiple complex interactions with both the contents of the vessel lumen and the cellular constituents of the vessel wall, where important mechanosensing and signal transduction processes guide maintenance of vascular tone and responses to stress [68]. Most of these interactions are mediated through nitric oxide (NO), a short-lived but potent vasodilator, produced by multiple NO synthase isoforms: neuronal (nNOS/NOS1), inducible (iNOS/NOS2) and endothelial (eNOS/NOS3) [94].

Endothelial dysfunction is both triggered by and propagates oxidative stress, leading to abnormal VSMC proliferation and/or apoptosis, increased endothelial permeability, and increased expression of inflammatory adhesion molecules, which altogether promote vascular dysfunction and pathological aortic wall remodelling [95]. Supporting this mechanism, an angiotensin II (Ang-II) infusion mouse model of TAA has shown increased endothelial-specific ROS associated with aortic dissection [96].

There is evidence of dysregulated **eNOS** and **NO** expression in genetically triggered TAA. In BAV-TAA, multiple studies have documented decreased eNOS in aortic tissue samples [97], including regional reductions in the greater curvature, which is the region most susceptible to haemodynamic stress and dilatation [98]. Decreased eNOS expression was also shown to correlate with increasing aortic diameter [99]. These human studies are supported by data from eNOS-knockout mouse models, which demonstrate a high prevalence of both BAV and aneurysm formation [100].

Uncoupling of eNOS has been observed in human BAV-TAA tissue [101] and a murine MFS-TAA model through a novel pathologic TGF-β/NOX4 axis [102]. Uncoupled eNOS is a hallmark feature of cardiovascular disease [103] and results in the production of ROS at the expense of NO, shifting its role from vasorelaxant to pro-oxidant and inducing ROS-mediated cellular damage [104]. Uncoupled eNOS has been correlated with AAA development [105, 106] and aortic rupture [106] in murine studies, and a similar mechanism is likely to occur in TAA pathogenesis. Notably, eNOS recoupling induced by infusion of dihydrofolate reductase (folic acid) was shown to ameliorated AAA formation [105] and attenuated Ang-II mediated vascular remodelling [106]. Causes of eNOS uncoupling are many but include excessive Ang-II, TGF-β, and ROS itself, which propagates a positive feedback loop of ROS-induced ROS formation [103]. Pharmacological modulation of the eNOS ‘redox switch’ is thus an active area of inquiry [103].

Inducible NOS (**iNOS**) is abnormally increased in human MFS-TAA tissue [107], and across a mixed cohort of various other forms of aortopathy [20]. iNOS is not expressed in the vasculature under physiological conditions but upregulated during oxidative stress and inflammation [13]. Results of a murine study suggest dysregulation in MFS may occur through a pathological Ang-II-Adamts1-NOS2/iNOS axis [107]. In addition to generation of NO, iNOS also produces peroxynitrite (ONOO^-^) which leads to further vascular damage and as such its dysregulation is implicated in several vascular [93] and systemic diseases [108].

## Future directions

There is extensive evidence of ROS dysregulation in genetically triggered TAA. It remains unknown whether this is a cause or consequence of TAA, and exactly what the pathological contribution of ROS is to aneurysm progression. If ROS do contribute to disease, their effective prevention or neutralisation requires an understanding of the specific pathological pathways and mediators involved, in addition to their physiological functions so that interventions do not cause further harm.

Identifying the specificity of ROS pathway mediators and cellular targets will always be plagued by the nature of ROS interactions, which occur at the atomic as opposed to macromolecular level [109], thus lacking target specificity, their short biological half-life thus impairing detection, and the redundancy of ROS scavengers facilitating their clearance, thus impeding pinpointing specific targets for manipulation. In light of these challenges, readers are directed to the most recent statement by the American Heart Association [110] which reviews the myriad available methods for detecting ROS and ROS-damage, including their strengths, limitations and suitability for different research objectives.

Antioxidant therapy involving either non-specific interventions (e.g. dietary [111]) or targeted approaches (e.g. xanthine oxidase inhibitors [112]), has been widely examined in cardiovascular disease and other disease contexts with both positive and negative findings, however, so far most have failed to confer benefit to all-cause mortality [113]. Similarly, while antioxidant interventions in animal TAA models have been shown to be effective, this has not been replicated in human clinical trials [13, 114, 115].

Efforts focusing on agents that block ROS production have proved more fruitful [62]. Selective inhibition of NOX enzymes (GKT136901/137831, Genkyotex; and VAS2870/3947, Vasopharm) have been shown to be well tolerated and efficacious in inflammatory diseases in clinical trials and are continuing to be assessed in cardiovascular and other diseases [62, 113]. Over 90 clinical trials in phase I-IV are underway assessing NRF-2 (nuclear factor (erythroid-derived 2) **–** like 2) activators in cardiovascular disease and its comorbid conditions [116, 117]. Drugs that target eNOS dysregulation in endothelial dysfunction are also being explored in small clinical trials, but data are so far inconclusive [103].

Other innovative methods being explored for high-precision ROS detection and targeted treatment in aneurysmal disease include the use of nanotechnology [118–120], *in vivo* fluorescent probes [119], and MRI-based methods [120]. The collaborative research efforts from pathology, redox physiology and biotechnology should continue to yield valuable insight into the nature of redox imbalance in genetically triggered TAA and will hopefully translate into clinical gains at the bedside to improve morbidity and mortality for patients.

## Conclusion

**Figure 1.**
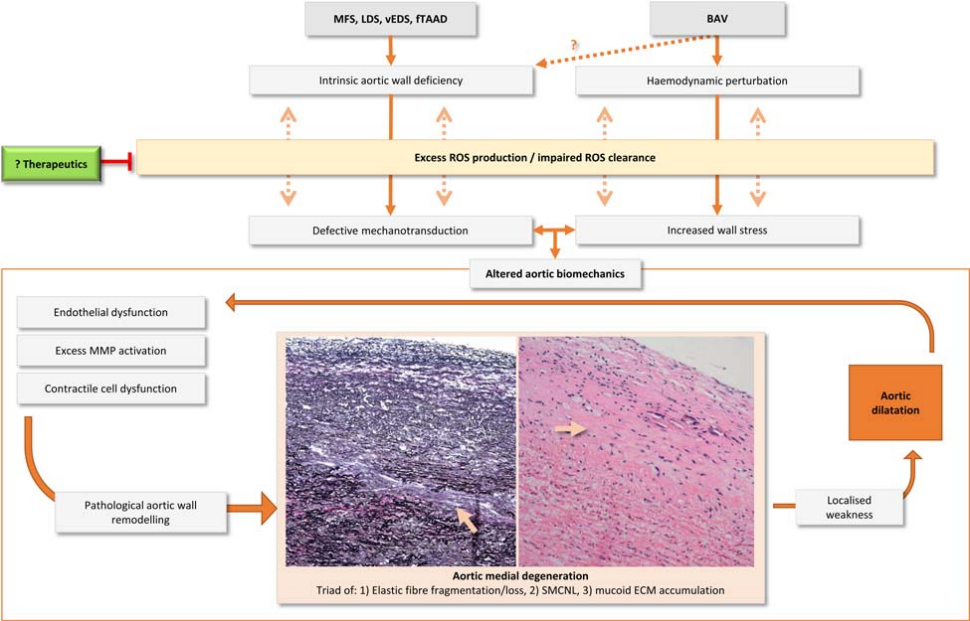
**Unanswered questions concerning the role of oxidative stress in genetically triggered TAA pathogenesis.** The relationship between oxidative stress and TAA development is likely due to altered aortic biomechanics, specifically due to intrinsic deficiencies in the aortic wall as in MFS, LDS, vEDS and fTAAD, or elevated wall stresses from turbulent flow as in BAV. Each render the aorta unable to adapt and instead undergo pathological remodelling leading to aneurysm formation. The specific contributions of redox pathway mediators to both oxidative stress and TAA pathogenesis are unknown. Therapeutics that can restore redox homeostasis may present a novel strategy to halt or reverse TAA development. MFS, Marfan syndrome; Loeys-Dietz syndrome; vEDS, vascular Ehlers Danlos syndrome; fTAAD, familial thoracic aortic aneurysm and dissection; BAV, bicuspid aortic valve; MMP, matrix metalloproteinase; SMCNL, smooth muscle cell nuclei loss; ECM, extracellular matrix.

There is an emerging body of evidence that confirms a role of oxidative stress in the pathogenesis of genetically triggered TAA but further research is required. End products of ROS-mediated cell damage present suitable candidates for biomarker development for staging and prognosis but the challenge lies in reliable, non-invasive quantitation. Therapeutic exploitation of ROS pathways may additionally prove effective in mitigating aneurysmal development and warrants continued investigation Figure 1.

## Ethical approval

This article does not contain any studies with human participants or animals performed by any of the authors.
